# Comprehensive Analyses of Masticatory Function in Maxillectomy Patients with Functioning Removable Prostheses: A Retrospective Cross-Sectional Study

**DOI:** 10.3390/jcm12155117

**Published:** 2023-08-04

**Authors:** Masahiro Kawasaki, Yoichiro Ogino, Ryoji Moroi, Yasunori Ayukawa

**Affiliations:** 1 Section of Fixed Prosthodontics, Division of Oral Rehabilitation, Faculty of Dental Science, Kyushu University, Fukuoka 812-8582, Japan; m-kawasaki@dent.kyushu-u.ac.jp (M.K.); rmoroi@dent.kyushu-u.ac.jp (R.M.); ayukawa@dent.kyushu-u.ac.jp (Y.A.); 2 Section of Implant and Rehabilitative Dentistry, Division of Oral Rehabilitation, Faculty of Dental Science, Kyushu University, Fukuoka 812-8582, Japan

**Keywords:** maxillectomy, masticatory function, oral functions, prostheses, tumor therapy

## Abstract

The aim of this retrospective cross-sectional study was to comprehensively assess masticatory function in maxillectomy patients with functioning removable prostheses. Their general and oral profiles, the measurement values of their oral functions, including masticatory function, and the history of tumor therapy were extracted from medical charts. The correlations of masticatory function with numerical data and the effects of tumor therapy-related factors on masticatory function were evaluated. In addition, a stepwise conditional logistic regression analysis was performed to identify the potential predictive factors comprehensively. The data from 55 maxillectomy patients revealed that the median value of masticatory function (138.0 mg/dL) was higher than the threshold (100.0 mg/dL) based on the concept of oral hypofunction. Moderate correlations of masticatory function with the number of remaining teeth, the number of functioning occlusal supports, and maximum occlusal force were found, as well as a weak correlation with maximum tongue pressure. These variables also showed statistically significant coefficients (*p* < 0.01). No significant effect of each tumor therapy-related factor on masticatory function was detected. A logistic regression analysis identified the number of functioning occlusal supports as a significant predictive factor. These results implied the crucial interactions of masticatory function with various factors and the specificities of maxillectomy patients.

## 1. Introduction

Maxillary resection, or “maxillectomy,” is conducted to ablate benign or malignant tumors as a radical treatment. Maxillectomy results in structural and functional alterations, and psychological distress. In particular, oral functions including mastication, deglutition, and speech are often impaired by maxillectomy, and reconstructive and/or prosthetic rehabilitation can be therapeutic options to restore oral functions [1,2,3,4,5]. Maxillectomy and the resulting deterioration of oral functions have been reported to be tightly associated with the patient’s health-related quality of life (HRQOL) and oral health-related quality of life (OHRQOL) [6,7,8], and oral rehabilitation is indispensable for overall activities of daily living.

Multiple oral functions and patient profiles have been reported to be associated with masticatory function in the healthy elderly population [9,10,11,12,13,14]. These studies suggested that masticatory function was associated with the number of present teeth, occlusal supports, occlusal force, and tongue pressure. In maxillectomy patients, similar findings have been reported in previous studies [1,5,15,16]. In addition to these factors, surgery-related factors should be considered to analyze their masticatory functions in maxillectomy patients, and previous studies demonstrated that some factors, including defect size and configuration, flap reconstruction, soft palate defects, and the number of remaining teeth, influenced masticatory function [17,18,19,20]. In the cases of maxillectomy resulting from tumor therapy, additional factors such as radiotherapy, chemotherapy, and sequential complications, which were regarded as tumor therapy-related factors, can be influential factors for HRQOL [21,22,23,24]. However, the studies that evaluated the association between these tumor therapy-related factors and masticatory function are still lacking. To understand the characteristics of masticatory function in maxillectomy patients, comprehensive assessments using a wide variety of factors would be desirable.

The aim of the present study was to comprehensively assess masticatory function in maxillectomy patients with functioning removable prostheses in surgically resected areas. In other words, tumor therapy-related factors such as ablative surgery, radiotherapy, and chemotherapy, patient profiles such as the number of remaining teeth and functioning occlusal supports, and the measurement values of their oral functions were adopted as potential predictive factors. Oral functions were evaluated using the methods for the diagnosis of “oral hypofunction,” which are well known in Japan [25,26]. Our hypothesis was that there were some significant factors affecting masticatory function in maxillectomy patients.

## 2. Materials and Methods

This retrospective cross-sectional study was conducted at the Department of Prosthodontics, Kyushu University Hospital, Fukuoka, Japan. This study was approved by our institutional review board for clinical research (approval number: 22163-01). This study was conducted in compliance with the Helsinki Declaration and the Strengthening the Reporting of Observational Studies in Epidemiology guidelines.

### 2.1. Study Subjects

The candidates for this study were maxillectomy patients with or without reconstructive surgeries who had visited our department from April 2014 to October 2022.

Inclusion criteria were as follows:

1. Patients with maxillectomy due to benign or malignant tumors;
2. Patients after prosthetic rehabilitations in surgically resected areas using removable prostheses without any implants and without any problems in their removable prostheses at the time of evaluation of their oral functions;
3. Patients with the data of their oral functions that had been evaluated when they had used their removable prostheses in surgically resected areas without any problems.

Exclusion criteria were as follows:

1. Patients with maxillectomy due to other diseases (except for tumors);
2. Patients without prosthetic rehabilitations, with prosthetic rehabilitations in surgically resected areas using fixed prostheses or implant-assisted prostheses, or with difficulties in the usage of their removable prostheses in surgically resected areas;
3. Patients without data on their oral functions.

### 2.2. Data Collection

The following data were extracted from a medical chart.

#### 2.2.1. Subjects’ Profiles

The data for subjects’ profiles include age, gender, primary tumor, and the number of remaining teeth and functioning occlusal supports. All data at the time of oral function evaluations were collected. The number of functioning occlusal supports was defined as the number of antagonistic occlusal contacts by natural teeth and fixed prostheses, including pontics. However, the occlusal contacts made by artificial teeth in removable prostheses were not counted. 

#### 2.2.2. Oral Functions

Oral functions were evaluated based on the concept of oral hypofunction [25,26]. These 5 functions and 2 additional functions (oral hygiene and swallowing function), totaling 7 functions, were used to diagnose oral hypofunction in Japan. When 3 or more functions are poorer than the threshold values, the patient is diagnosed with oral hypofunction [25,26]. However, in this study, 4 functions (except for masticatory function) that have been reported to be associated with masticatory functions in previous studies were used, and a total of 5 functions were extracted from the medical charts and analyzed. All functions were evaluated after the adjustment of their removable prostheses, meaning when the subjects could use them without any problems. 

**Figure 1:**
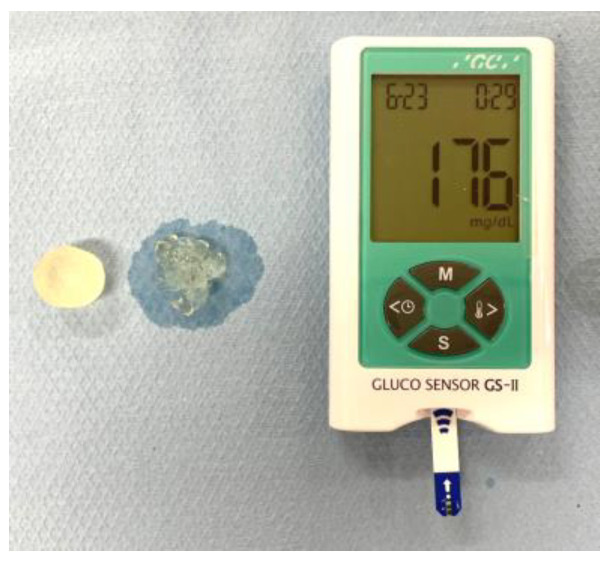
Gummy jellies before and after crushing, and a measurement device.

**Figure 2:**
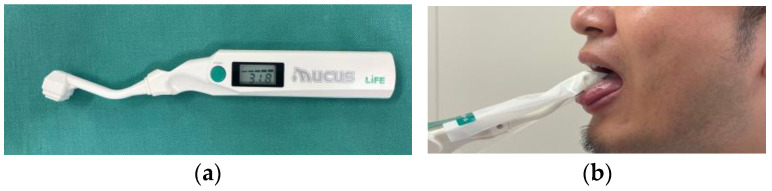
Measurement of oral moisture: (**a**) a measurement device (Mucus); (**b**) measurement using this device.

**Figure 3:**
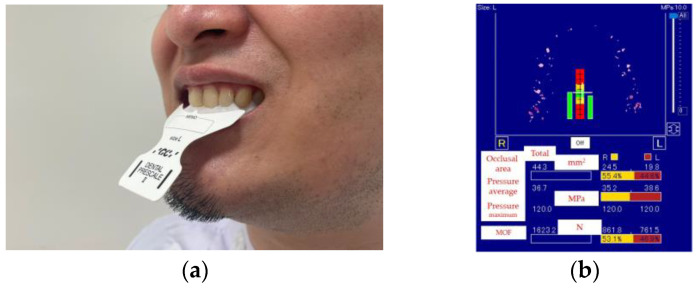
Measurement of maximum occlusal force (MOF): (**a**) clenching a pressure-sensitive sheet in the intercuspal position; (**b**) the results.

**Figure 4:**
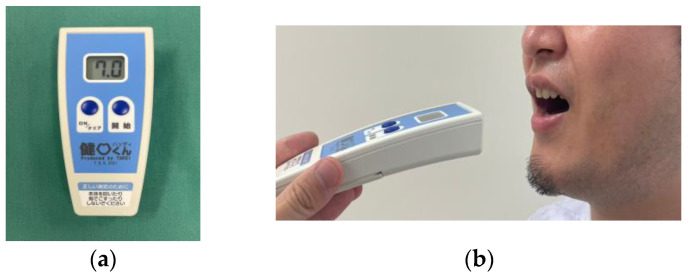
Measurement of tongue–lip motor function: (**a**) a measurement device (Kenkokun); (**b**) measurement using this device.

**Figure 5:**
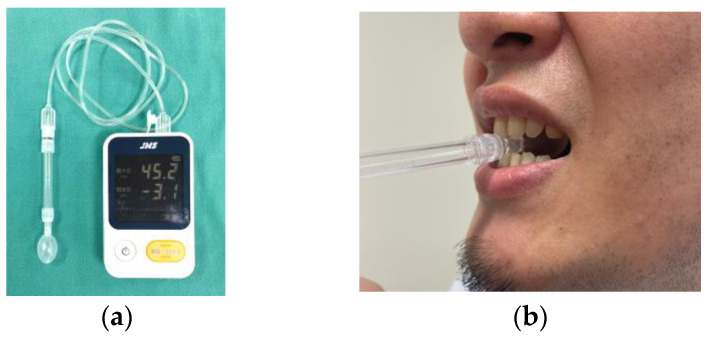
Measurement of maximum tongue pressure: (**a**) a measurement device (TPM-01); (**b**) measurement using this device.

Oral functions and the measurement methods were as follows:

1. Masticatory function: masticatory function was defined by the glucose concentration in 10 mL water from the particles of crushed gummy jelly (2 g) after 20 s voluntary chewing. Commercially available devices (Glucoram (a gummy jelly) and Gluco Sensor GS-II, GC Co., Tokyo, Japan) were adopted as described previously [5,15], and images are shown in Figure 1. The threshold value was defined as 100 mg/dL [25,26].

2. Oral moisture (OM): OM was measured using an oral moisture checker (Mucus, Life Co., Ltd., Saitama, Japan) (Figure 2). Briefly, a tip of this device with a disposal cover was put on the tongue for a few seconds, and the measurement value was presented. The threshold value was defined as 27.0, and a lower value suggested oral dryness [25].

3. Maximum occlusal force (MOF): A pressure-sensitive sheet (Dental Prescale II, GC Co., Tokyo, Japan) was used to record MOF after maximum clenching and was analyzed with a dedicated software (Bite Force Analyzer, GC Co., Tokyo, Japan) (Figure 3). The threshold value was defined as 500 N [5,26].

4. Tongue–lip motor function; oral diadochokinesis (ODK): tongue–lip motor function was defined by ODK (Figure 4). The repetitive syllables /pa/, /ta/, and /ka/ for 5 s were counted using a commercially available device (Kenkokun Handy, Takei Scientific Instruments Co., Ltd., Niigata, Japan) to evaluate lip motor function, the anterior and posterior regions of tongue motor function, respectively. The threshold value was defined as 6 times per second [25,26].

5. Maximum tongue pressure (MTP): MTP was measured using a commercially available device (JMS tongue pressure measuring device TPM-01, JMS Co., Ltd., Hiroshima, Japan) (Figure 5). Briefly, the compression against an inflated balloon produced by tongue elevation was defined as MTP. The threshold value was defined as 30 kPa [25,26].

#### 2.2.3. History of Their Tumor Therapy

The history of tumor therapies includes the extent of the resected defect (unilateral or bilateral defect), the existence or non-existence of soft palate resection, oronasal and oroantral communication, radiotherapy, chemotherapy, and neck dissection.

### 2.3. Statistical Analyses

Numerical data from medical charts are described as median values and interquartile ranges (IQRs). Spearman correlation coefficients (ρ) were calculated between masticatory function and other numerical data (age, the numbers of remaining teeth and functioning occlusal supports, and the values of oral functions) to validate the possible correlations. The Wilcoxon rank sum test was used to compare masticatory function between the subjects with and without the histories of their tumor therapies. A stepwise conditional logistic regression analysis was performed to verify the potential predictive factors using multivariable-adjusted odds ratios (ORs) and 95% confidence intervals (CIs). Numerical data were classified into 2 groups according to the median values (the numbers of remaining teeth and functioning occlusal supports) or the threshold value of each factor for a stepwise conditional logistic regression analysis and calculation of odds ratios. The JMP16 software (SAS Institute Inc., Cary, NC, USA) was used for all statistical analyses, and a value of *p* < 0.05 was considered statistically significant.

## 3. Results

### 3.1. Study Subjects and Numerical Data

The present study included 55 subjects (male: 27; female: 28). They had received maxillectomy due to adenocarcinoma (2 subjects), adenoid cystic carcinoma (4 subjects), mucoepidermoid carcinoma (2 subjects), squamous cell carcinoma (38 subjects), chondrosarcoma (1 subject), oral melanoma (1 subject), pleomorphic adenoma (1 subject), ameloblastoma (1 subject), and 5 subjects had been unknown (no record). Maxillectomies were classified into 3 categories: limited maxillectomy or partial resection (47 subjects), subtotal maxillectomy (4 subjects), and total maxillectomy (4 subjects), and extents of resected defects were classified into two categories, unilateral (31 subjects) and bilateral (24 subjects), which were adopted as tumor therapy-related factors. The median value and IQR of each numerical variable are shown in Table 1. The present study revealed that the median values of oral functions that exceeded the threshold values were masticatory function (median and IQR: 138.0 and 86.0–188.0) and OM (median and IQR: 28.2 and 26.4–29.5).

### 3.2. Correlations between Masticatory Function and Other Numerical Data

The correlations between masticatory function and other numerical data are presented in Table 2. The present study indicated moderate correlations of masticatory function with the number of remaining teeth, the number of functioning occlusal supports, and MOF (0.40 ≤ ρ < 0.70) and weak correlations with MTP (ρ = 0.39), and these variables also showed statistically significant coefficients (*p* < 0.01).

### 3.3. Effects of Tumor Therapy-Related Factors on Masticatory Function

The effects of tumor therapy-related factors on masticatory function were evaluated by comparing two groups (with and without therapy; Wilcoxon rank sum test). Although the median values of masticatory function in the subjects with soft palate resection, oronasal and oroantral communication, chemotherapy, and neck dissection, meaning more intervention groups, were higher compared to the subjects without these interventions, unexpectedly, these analyses revealed that no tumor-related therapies induced significant differences in masticatory function (Table 3).

### 3.4. A Comprehensive Analysis of Masticatory Functions in Maxillectomy Patients

Including all factors analyzed in this study (subjects’ profiles, oral functions, and tumor therapy-related factors), a stepwise conditional logistic regression analysis was conducted. As described above, all subjects were classified into two groups according to median values: the threshold value of each oral function and the history (with or without) of each tumor therapy. The factors selected in a stepwise method were the number of functioning occlusal supports and OM, and multivariable-adjusted ORs are shown in Table 4. This analysis demonstrated that the number of functioning occlusal supports was a significant predictive factor in this study.

## 4. Discussion

Mastication is the process of forming a bolus for deglutition and digestion using teeth, saliva, tongue, masticatory muscles (occlusal force), and the neuromuscular system (mandibular motor function) [27]. Previous studies suggested that these factors played important roles in performing satisfactory mastication [9,10,11,12,13,14], and mastication is a complex process anatomically and physiologically. 

Tumor therapies, including surgical resection, radiotherapy, and chemotherapy, cause multiple complications. In particular, maxillectomy for oral or head and neck tumors results in the loss of anatomical structures and impaired physiologic functions. Masticatory function is one of the most affected functions by maxillectomy, and surgical reconstruction and/or prosthetic rehabilitation are necessary to restore this function. However, maxillectomy patients who had received multiple tumor therapies might possess various conditions [28], and this study was planned to identify the factors influencing masticatory function.

At first, oral functions were evaluated based on the concept of oral hypofunction [25,26]. The subjects in this study demonstrated that the median values of masticatory function and OM exceeded their thresholds, although other functions had comparatively lower values. Masticatory function is associated with various factors, implying that the cooperativeness of several factors might permit better masticatory function. Some previous studies showed that masticatory function was comparable between maxillectomy patients and various patients without maxillectomy (healthy dentate patients, healthy edentulous patients, and partially edentulous patients) [2,15,29,30]. In addition, one report analyzed masticatory function using the same methods and threshold values, and a similar finding was confirmed [5]. Although we understand that the difficulty of rehabilitation depends on the individual conditions, these results suggest that masticatory function in maxillectomy patients could be restored with prosthetic rehabilitation to some extent.

Second, the correlations between masticatory function and numerical data, including the numbers of remaining teeth and functioning occlusal supports, and oral functions were analyzed using Spearman rank correlations. These analyses showed that the numbers of remaining teeth, functioning occlusal supports, and MOF exhibited moderate correlations with masticatory function. In addition, MTP exhibited a weak correlation. All correlations were statistically significant. These results were consistent with previous studies [1,3,5,15,16,17,19,20,29]. However, these previous studies did not show significant correlations between masticatory function and all these variables, which was a distinctive finding of this study. The previous studies also showed that these variables played significant roles in masticatory function in the subjects without maxillectomy [9,10,11,12,13,14], and this result suggested the regulation of masticatory function in maxillectomy patients would be similar to that of the subjects without maxillectomy. 

Third, the effects of tumor therapy-related factors on masticatory function were evaluated. The present study indicated that no significant difference between masticatory function in the subjects with and without each therapy was identified, although some groups with more interventions showed higher median values of masticatory function. According to the previous studies, some tumor therapy-related factors, such as the extent of the resected defect [5,15,17,19,29], soft palate resection [3], and oronasal and oroantral communication [4,6,19], were evaluated. The results of this study (no significant effect of each therapy on masticatory function) suggested a complex regulation of mastication by multiple factors. In fact, the specific effects of some factors on masticatory function were observed at each subject level. However, the present study did not reveal any overall effect of each factor on masticatory function statistically, implying that the interactions with the variables, including oral status and oral functions, would regulate masticatory functions. 

Lastly, based on the presence of multiple variables and potential interactions, a stepwise conditional logistic regression analysis was performed to assess the predictive factors, including all numerical variables and tumor therapy-related factors, which were nominal variables. This analysis identified the number of functioning occlusal supports as a significant predictive factor. It would be reasonable if a greater number of functioning occlusal supports acted positively on masticatory function. As described above, some tumor therapy-related factors could have been predictive factors in previous studies [3,4,5,6,15,17,19,29]. All subjects in this study were rehabilitated with well-functioning removable prostheses, and this inclusion criterion might influence the results despite their diverse oral conditions and various tumor therapies. Furthermore, the interactions among multiple variables might play crucial roles in masticatory functions, resulting in a comparatively higher masticatory function (the median value was higher than the threshold value). Although, for example, the immediate complications of radiotherapy and/or chemotherapy, such as mucositis, might affect oral functions [31], the effects of the late complications induced by these therapies on masticatory function are still unknown. Masticatory function was evaluated after prosthetic rehabilitation and comfortable usage, meaning immediate and early symptoms had been resolved and the oral conditions would be stable. These conditions might be reflected in the results of this study, which show that the number of functioning occlusal supports was the only factor that statistically influenced masticatory function among multiple variables. A recent study evaluated the correlation between masticatory function and other factors comprehensively, and they suggested that a history of radiation therapy, maximum bite force, number of remaining teeth, tongue pressure, and part of the tongue–lip motor function were related to masticatory function [32]. It seemed likely that they could show some similarities to the results of the present study. The different points of the present study were the study subjects and the number and features of the explanatory variables. The subjects in their study included maxillectomy and mandibulectomy patients. Surgical intervention for mandibulectomy may affect mandibular movement and tongue motor function more severely compared to maxillectomy. Furthermore, their study adopted time-dependent variables (periods after surgery and the delivery of dentures) as explanatory variables. The present study used other tumor therapy-related factors such as oronasal and oroantral communication, chemotherapy, and neck dissection, which were regarded as major factors in tumor therapy. The differences between these explanatory variables would influence the results. Considering the limitations of this study, including confounding factors, further studies using more subjects under as similar conditions as possible would be necessary to evaluate their masticatory function comprehensively. However, the results of this study would be important to understand masticatory function and its specificity in maxillectomy patients with various backgrounds.

## 5. Conclusions

The present study comprehensively evaluated masticatory function and its influential factors in maxillectomy patients. Each individual factor, such as the number of remaining teeth and functioning occlusal supports, MOF, and MTP, showed a significant correlation with masticatory function. No significant effect of each tumor therapy-related factor on masticatory function was detected. A stepwise conditional logistic regression analysis suggested that the number of functioning occlusal supports plays a crucial role among potential variables. These results implied the specificities of maxillectomy patients.

## Figures and Tables

**Table 1:** Numerical data.

Variables	Median (IQR)
Age	71.0 (64.0–77.0)
Number of remaining teeth	18.0 (12.0–21.0)
Number of functioning occlusal supports	6.0 (2.0–8.0)
Masticatory function (mg/dL)	138.0 (86.0–188.0)
OM	28.2 (26.4–29.5)
MOF (N)	348.7 (173.4–547.0)
ODK/pa/(times/s)	4.8 (3.8–5.6)
ODK/ta/(times/s)	4.6 (3.6–5.4)
ODK/ka/(times/s)	4.4 (3.2–5.2)
MTP (kPa)	26.9 (21.8–31.5)

IQR: interquartile range, OM: oral moisture, MOF: maximum occlusal force, ODK: oral diadochokinesis, MTP: maximum tongue pressure.

**Table 2:** Spearman correlation coefficient between masticatory function and other numerical data.

Variables	Spearman Correlation Coefficient (ρ)	*p*-Value
Age	−0.20	0.47
Number of remaining teeth	0.47	<0.01 *
Number of functioning occlusal supports	0.45	<0.01 *
OM	0.23	0.13
MOF (N)	0.54	<0.01 *
ODK/pa/ (times/s)	0.20	0.10
ODK/ta/ (times/s)	0.22	0.06
ODK/ka/ (times/s)	0.10	0.31
MTP (kPa)	0.39	<0.01 *

OM: oral moisture; MOF: maximum occlusal force; ODK: oral diadochokinesis, MTP: maximum tongue pressure. * Statistically significant: *p* < 0.01.

**Table 3:** Comparisons of masticatory function between the subject groups classified according to tumor therapy-related factors.

Tumor Therapy-Related Factors	(+ or −): Number of Subjects Masticatory Function: Median (IQR) (mg/dL)		*p*-Value *
Extent of resected defect	Bilateral: 24	Unilateral: 31	0.47
	114.5 (87.3–168.0)	147.0 (75.0–222.0)	
Soft palate resection	(+): 24	(−): 31	0.06
	153.0 (101.0–223.2)	120.0 (70.0–184.0)	
Oronasal and oroantral communication	(+): 40	(−): 15	0.07
	146.0 (97.0–198.5)	107.0 (56.0–169.0)	
Radiotherapy	(+): 25	(−): 30	0.15
	121.0 (69.0–165.0)	147.0 (92.5–224.3)	
Chemotherapy	(+): 22	(−): 33	0.73
	141.5 (91.3–166.0)	136.0 (81.0–212.0)	
Neck dissection	(+): 18	(−): 37	0.70
	149.5 (73.0–232.3)	132.0 (88.5–186.0)	

IQR: Interquartile Range; * Results of Wilcoxon rank sum test.

**Table 4:** Results of a stepwise conditional logistic regression analysis.

Objective Variable	Explanatory Variables	OR	95% CI	*p*-Value
Masticatory function	Number of functioning occlusal supports	3.42	1.06–12.07	0.04 *
	OM	2.35	0.70–8.14	0.16

OM: oral moisture; OR: odds ratio; CI: confidence interval. * Statistically significant: *p* < 0.05.

## Data Availability

The datasets used and/or analyzed during the current study are available from the corresponding author upon reasonable request.
